# Downregulation of KPNA2 in non-small-cell lung cancer is associated with Oct4 expression

**DOI:** 10.1186/1479-5876-11-232

**Published:** 2013-09-26

**Authors:** Xiao-Lei Li, Lan-Ling Jia, Mu-Mu Shi, Xin Li, Zhong-Hua Li, Hui-Feng Li, En-Hua Wang, Xin-Shan Jia

**Affiliations:** 1 Department of Pathology, the First Affiliated Hospital and College of Basic Medical Sciences of China Medical University, Shenyang 110001, China; 2 Department of Pathology, College of Basic Medical Sciences, Haerbin Medical University-Daqing, Daqing 163319, China; 3 Department of Pharmacology, College of Life Science and Biopharmaceutics of Shenyang Pharmaceutical University, Shenyang 110001, China; 4 Department of Critical Care Medicine, Daqing People’s Hospital, Daqing 163319, China; 5 Department of Pathology, Daqing General Hospital Group Oilfield General Hospital, Daqing 163319, China

**Keywords:** Non-small-cell lung cancer, Oct4, KPNA2, Nucleocytoplasmic transport

## Abstract

**Background:**

Oct4 is a major transcription factor related to stem cell self-renewal and differentiation. To fulfill its functions, it must be able to enter the nucleus and remain there to affect transcription. KPNA2, a member of the karyopherin family, plays a central role in nucleocytoplasmic transport. The objective of the current study was to examine the association between Oct4 and KPNA2 expression levels with regard to both the clinicopathological characteristics and prognoses of patients with non-small-cell lung cancer (NSCLC).

**Methods:**

Immunohistochemistry was used to detect the expression profile of Oct4 and KPNA2 in NSCLC tissues and adjacent noncancerous lung tissues. Real-time polymerase chain reaction and western blotting were used to detect the mRNA and protein expression profiles of Oct4 and KPNA2 in lung cancer cell lines. Small interfering RNAs were used to deplete Oct4 and KPNA2 expressions. Double immunofluorescence was used to detect Oct4 expression in KPNA2 knockdown cells. Co-immunoprecipitation was used to detect the interaction of Oct4 and KPNA2.

**Results:**

Oct4 was overexpressed in 29 of 102 (28.4%) human lung cancer samples and correlated with differentiation (*P* = 0.002) and TNM stage (*P* = 0.003). KPNA2 was overexpressed in 56 of 102 (54.9%) human lung cancer samples and correlated with histology (*P* = 0.001) and differentiation (*P* = 0.045). Importantly, Oct4 and KPNA2 expression levels correlated significantly (*P* < 0.01). Expression of Oct4 and KPNA2 was associated with short overall survival. In addition, depleting Oct4 and KPNA2 expression using small interfering RNAs inhibited proliferation in lung cancer cell lines. Real-time polymerase chain reaction and western blotting analysis indicated that reduction of KPNA2 expression significantly reduced mRNA and nucleoprotein levels of Oct4. Double immunofluorescence analysis revealed that nuclear Oct4 signals were reduced significantly in KPNA2 knockdown cells. Co-immunoprecipitation experiments revealed that KPNA2 interacts with Oct4 in lung cancer cell lines.

**Conclusion:**

Oct4 and KPNA2 play an important role in NSCLC progression. Oct4 nuclear localization may be mediated by its interaction with KPNA2.

## Background

Oct4, also known as POU5F1 (POU domain, class 5, transcription factor 1), is encoded by the *POU5F1* gene in humans [1]. It is a homeodomain transcription factor of the POU family. Oct4 is normally found in totipotent or pluripotent stem cells of pregastrulation embryos and is essential to maintain their self-renewal [2]. Downregulation of Oct4 results in loss of stem cells [3]. The Oct4 transcription factor can be considered a master regulator of maintenance and differentiation in pluripotent cells. Previous studies have indicated an involvement of Oct4 in tumorigenicity and malignancy of lung cancers [4]. Oct4 might be a biomarker for assessing the prognosis of hepatocellular carcinoma and gastric cancer [5,6]. For patients with hypopharyngeal squamous cell carcinoma, Oct4 expression is an independent predictive factor [7]. Oct4 expression has also been observed in human breast cancer stem-like cells, suggesting an involvement in self-renewal and tumorigenesis [8]. Three isoforms of Oct4 have been identified – Oct4A, Oct4B, and Oct4B1. Oct4A is primarily localized in the nucleus, whereas Oct4B and Oct4B1 primarily reside in the cytoplasm. Currently, only Oct4A has been proven to regulate pluripotency [9].

Transcription factors need to enter and remain in the nucleus to fulfill their functions. The Oct4 transcription factor homeodomain signature structure is important for both DNA binding and nucleocytoplasmic trafficking [10,11]. The importin transport system provides the machinery for nucleocytoplasmic transport. Nuclear protein transport is a complex process and aberrant expression of nuclear transport factors may result in profound deregulation of gene transcription [12]. KPNA2 (importin α1), a member of the karyopherin (importin) family, plays a central role in nucleocytoplasmic transport [13]. Together with importin-β, the KPNA2-bound cargo protein forms a ternary complex, which interacts with the nuclear pore complex and translocates into the nucleus, guided by a nuclear localization signal [14]. A previous study showed that KPNA2 is mainly expressed in undifferentiated embryonic stem (ES) cells, and nuclear import of Oct4 is mediated by KPNA2 in mouse ES cells during differentiation into neurons [15]. Oct4 and KPNA2 are reported to have a strong interaction in ES cells [16]. In addition, KPNA2 was identified as a potential biomarker for non-small-cell lung cancer (NSCLC) by integration of the cancer cell secretome and tissue transcriptome [17]. For lung cancer, however, very few reports have mentioned the two genes. This current study provides direct evidence for the interaction between Oct4 and KPNA2 and demonstrates that KPNA2 may contribute to Oct4 nuclear translocation in lung cancer.

## Methods

### Patient populations and clinical specimens

The Ethics Committee of the China Medical University approved this study. One hundred and two NSCLC samples and the corresponding normal lung tissues were obtained from the First Affiliated Hospital of China Medical University during 2004–2007. Tissues were formalin fixed and paraffin embedded. Before the operation, the patients had not received chemotherapy or radiotherapy. Histological types and differentiation degrees were categorized by the 2004 WHO standards, and TNM were classified according to the 2009 UICC TNM classification. Relevant clinical and pathological features (Table 1) were obtained from the patients’ files and/or by telephone interviews with the patient or their relatives.

**Table 1:** The expression of Oct4 and KPNA2 and their relationships with clinicopathological characteristics

**Features**	**No.**	**Nuclear Oct4 immunoreactivity**			**Nuclear KPNA2 immunoreactivity**		
		**Negative**	**Positive (%)**	***P***	**Negative**	**Positive (%)**	***P***
Age (years)							
<60	50	36(72.0)	14(28.0)	0.925	25(50.0)	25(50.0)	0.329
≥60	52	37(71.2)	15(28.8)		21(40.4)	31(59.6)	
Gender							
Male	59	42(71.2)	17(28.8)	0.920	28(47.5)	31(52.5)	0.574
Female	43	31(72.1)	12(27.9)		18(41.9)	25(58.1)	
Smoke							
Yes	52	36(69.2)	16(30.8)	0.593	22(42.3)	30(57.7)	0.563
No	50	37(74.0)	13(26.0)		24(48.0)	26(52.0)	
Histology							
Adenocarcinoma	60	41(68.3)	19(31.7)	0.387	35(58.3)	25(41.7)	0.001*
Squamous cell	42	32(72.6)	10(23.8)		11(26.2)	31(73.8)	
Carcinoma							
Differentiation							
Well	38	34(89.5)	4(10.5)	0.002*	22(57.9)	16(42.1)	0.045*
Moderate-Poor	64	39(60.9)	25(39.1)		24(37.5)	40(62.5)	
TNM stage							
I/II	55	46(83.6)	9(16.4)	0.003*	29(52.7)	26(47.3)	0.093
III/IV	47	27(57.4)	20(42.6)		17(36.2)	30(63.8)	
Angiolymphatic invasion							
No	57	44(77.2)	13(22.8)	0.156	25(43.9)	32(56.1)	0.777
Yes	45	29(64.4)	16(35.6)		21(46.7)	24(53.3)	

*P < 0.05.

### Immunohistochemistry (IHC) and scoring

The paraffin-embedded tissues were cut into 5-μm sections, placed on slides, and baked at 60°C for 120 min. The sections were de-paraffinized with xylenes and rehydrated. They were subjected to heat-induced antigen retrieval using citrate buffer (10 mM, pH 6.0) in a pressure cooker for 2 min and then cooled to room temperature for 20 min. The sections were treated with 3% hydrogen peroxide in methanol to quench the endogenous peroxidase activity, followed by incubation with normal serum to block nonspecific binding. The slides were then incubated with an anti-Oct4 antibody (1:100) (Santa Cruz Biotechnology, Inc., Santa Cruz, CA, USA) and an anti-KPNA2 antibody (1:100) (Santa Cruz Biotechnology) overnight at 4°C, respectively. As a negative control for the staining procedure, the primary antibody was replaced with PBS. The second antibody was from an SP reagent kit (Zhongshan Biotechnology Company, Beijing, China). After washing, the tissue sections were treated with a biotinylated anti-mouse second antibody, followed by further incubation with streptavidin-horseradish peroxidase complex for 15 min. The sections were stained with diaminobenzidine and then counterstained with hematoxylin. The stained slides were reviewed and scored independently by two pathologists. The proportion (0: none; 1: < 25%; 2: 25–50%; 3: 51–75%; and 4: > 75%) and intensity (0, none; 1, weak; 2, intermediate; and 3, strong) scores were added to obtain a total score, which ranged from 0 to 7. Specimens were categorized into one of two groups according to their overall scores: (1) negative expression, < 4 points; (2) positive expression, 4–7 points.

### Cell culture

Human lung cancer cell lines A549, LTE, 1299, and H460 were obtained from the American Type Culture Collection (Manassas, VA, USA). LK2 was obtained from the Japanese Cancer Research Resources Bank (Tokyo, Japan), SPC cells were obtained from the CCTCC (Chinese Center of Typical Culture Conserve, Wuhan, P.R. China). The cells were cultured in RPMI 1640 medium (Invitrogen, Carlsbad, CA, USA) containing 10% fetal calf serum (Invitrogen), 100 IU/ml penicillin (Sigma, St. Louis, MO, USA), and 100 μg/ml streptomycin (Sigma).

### Western blot analysis

Total proteins from cell lines were extracted in lysis buffer (Thermo Fisher Scientific, Rockford, IL, USA) and quantified using the Bradford method. Each extracted protein sample (50 μg) was separated by sodium dodecyl sulfate-polyacrylamide gel electrophoresis. After transferring to a polyvinylidene fluoride membrane, the membrane was incubated overnight at 4°C with the mouse monoclonal antibody against Oct4 (1:500, Santa Cruz Biotechnology), KPNA2 (1:500, Santa Cruz Biotechnology), β-actin (1:1000, Santa Cruz Biotechnology), or anti-lamin-B (1:500 Santa Cruz Biotechnology). After incubation with peroxidase-coupled anti-mouse IgG at 37°C for 2 h, the protein bands were visualized using ECL (Pierce, Rockford, IL, USA) and detected using the BioImaging Systems (UVP Inc., Upland, CA, USA). The relative amounts of protein were calculated with reference to the amount of β-actin protein.

### Quantitative real-time polymerase chain reaction (PCR)

Total cellular RNA was extracted from cells using the RNeasy Mini kit from Qiagen (Hilden, Germany). Reverse transcription of 1 μg of RNA was performed using a high-capacity cDNA RT kit (Applied Biosystems, Foster City, CA, USA), following the manufacturer’s instructions. Quantitative real-time PCR was performed using a SYBR Green PCR master mix (Applied Biosystems) in a total volume of 20 μl on a 7900HT fast Real-Time PCR System (Applied Biosystems). A dissociation step was performed to generate a melting curve for confirmation of amplification specificity. β-actin was used as the reference gene. The relative levels of gene expression were represented as △Ct = Ct gene-Ct reference, and the fold change of gene expression was calculated by the 2-△△Ct method. Experiments were repeated in triplicate. The primer sequences were as follows: KPNA2 forward, 5′-CTGGGACATCAGAACAAACCAAG- 3′, KPNA2 reverse, 5′-ACACTGAGCCATCACCTGCAAT- 3′; Oct4 forward, 5′- CGCAAGCCCTCATTTCAC-3′; Oct4 reverse, 5′- CATCACCTCCACCACCTG-3′; β-actin forward, 5′-ATAGCACAGCCTGG ATAGCAACGTAC-3′, β-actin reverse, 5′-CACCTTCTACAATGAGCTGCGTGTG- 3′.

### Immunofluorescence staining

Cells were placed into 96-well plain bottom plates, and fixed in 4% formaldehyde, permeabilized in 0.5% Triton X-100, and washed with PBS containing 1% of BSA (FACS buffer). After incubation with primary antibodies at 4°C overnight, the wells were washed with PBS three times. Appropriate secondary antibodies were added and incubated at 37°C for 30 min. The cells were then stained with 4′, 6-diamidino-2-phenylindole (DAPI) for 15 min. Specimens were examined using an epi-illumination fluorescence microscope BX50 (Olympus, Tokyo, Japan).

### Knockdown of Oct4 and KPNA2 expression with small interfering RNA

Cells (1 × 10^4^) were transfected with HiPerfect transfection reagent (Giagen, Germany), applying a combination of two sequence-validated, and knockdown-warranted siRNAs: KPNA2-siRNA (20 nM, each, 5′ -GCUCCUGCAUCAUGAUGAUTT-3′ and 5′-AUCAUCAUGAUGCAGGAGCTT-3′) and Oct4-siRNA (20 nM, each, 5′-CUGGGACACAGUAGAUAGATT-3′ and 5′-UCUAUCUACUGU GUCCCAGTT-3′), according to the manufacturer’s instructions (GenePharma Co., Ltd, Germany). After 96 h of treatment, the cells were split and re-transfected with siRNAs to guarantee efficient KPNA2 and Oct4 knockdown. A commercial GAPDH-siRNA served as a positive control (GenePharma). A green fluorescent protein-tagged, NCsiRNA (GenePharma) was used as an efficiency control and as a control for unspecific side effects. At 72 h after transfection, cell lysates were prepared for western blotting to determine the efficiency of gene expression ablation.

### Cell proliferation assay

Cell proliferation was assayed using a Cell Counting Kit-8 solution (Dojindo, Gaithersburg, MD, USA), according to the manufacturer’s protocol. Briefly, cells were seeded at a concentration of 2 × 10^3^ cells/100 μl/well in 96-well culture plates and treated with 10 μl/well of Cell Counting Kit-8H solution during the last 4 h of the culture. The optical density of the wells was measured at 450 nm using a microplate reader (Model450, Bio-Rad, Hercules, CA, USA).

### Sphere formation assay

Cells (1 × 10^3^) were seeded into 6-cm dishes and maintained in growth medium. After 10 days, colonies were fixed with 80% methanol and stained with Giemsa (20 min.). Colonies were photographed and counted. The number of colonies with more than 50 cells was recorded.

### Co-immunoprecipitation (CoIP)

Proteins were extracted with cellular lysis buffer. Equal amounts of protein were incubated with the KPNA2 specific antibody immobilized onto protein G-beads for 1 h at 4°C with gentle rotation. Beads were washed extensively with lysis buffer, boiled, and microcentrifuged. Proteins were detected with the anti-Oct4 antibody by western blotting.

### Statistical analysis

SPSS version 13.0 for Windows was used for all analyses. The Chi-square test was used to compare positive staining rates between subgroups. Kaplan–Meier curves were plotted for survival analysis, and a log-rank test was performed based on the differences. The Student’s t-test was used to compare other data. A *P*-value of < 0.05 was considered statistically significant, and a *P*-value of < 0.01 was considered strongly statistically significant.

## Results

### Overexpression of Oct4 and KPNA2 in NSCLC tissues

**Figure 1:**
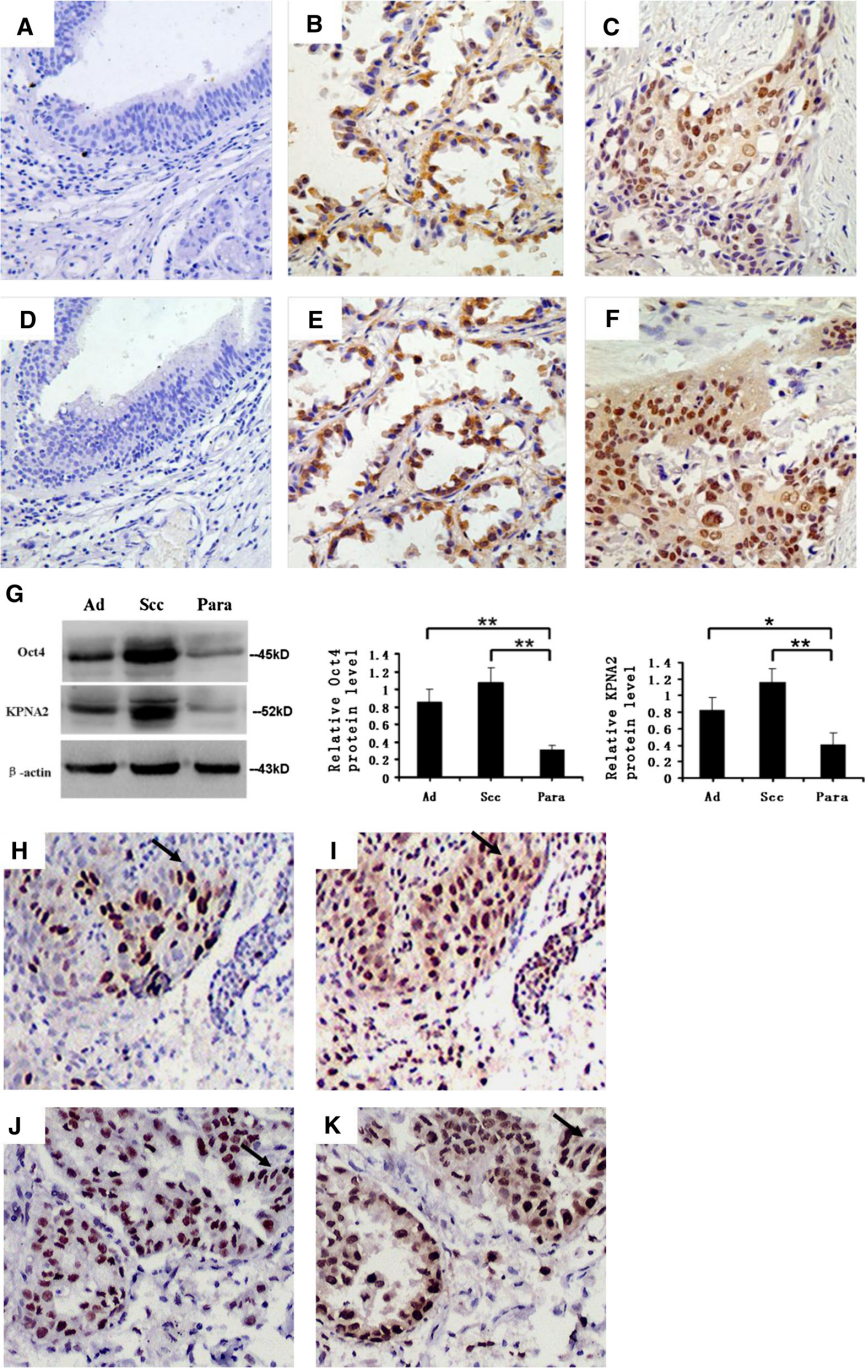
**Expression of Oct4 and KPNA2.** Immunohistochemical staining for Oct4 and KPNA2 expression in cancerous tissues and their corresponding paracancerous tissues. Brown grains represent a positive signal. The positive expression site of Oct4 and KPNA2 was mainly in the nucleus of tumor cells. Oct4 negative expression in paracancerous tissue (Para) **(A)**; Oct4 positive expression in lung adenocarcinoma (Ad) **(B)** and lung squamous cell carcinoma (SCC) **(C)**; KPNA2 negative expression in Para **(D)**; KPNA2 positive expression in lung Ad **(E)** and lung SCC **(F)**. Western blot analysis of the expression of Oct4 and KPNA2 in lung Ad, lung SCC, and Para **(G)**. Each bar represents the mean ± SD of three independent experiments. *P < 0.05, **P < 0.01. Immunohistochemical staining for Oct4 **(H)** and KPNA2 **(I)** in a case of lung SCC. Immunohistochemical staining for Oct4 **(J)** and KPNA2 **(K)** in a case of lung Ad. Original magnification, 400 × .

We investigated Oct4 and KPNA2 protein expression in 102 human NSCLC cancerous and precancerous tissue samples by IHC. Overall, for both Oct4 and KPNA2, positive immunostaining was only observed in the nuclei of the cancer cells, whereas all the precancerous tissues showed negative staining (Figure 1A–F). Among the 102 cancerous tissue samples, 29 (28.4%) showed positive staining for Oct4, and 56 (54.9%) showed positive staining for KPNA2. Twenty-six (25.5%) cases showed both Oct4 and KPNA2 positive staining; 43 (42.2%) cases were negative for both Oct4 and KPNA2 staining; and 33 (32.3%) cases showed either Oct4 or KPNA2 positive staining. The western blotting results agreed with the IHC results. The protein expressions of Oct4 and KPNA2 in NSCLC tissues were significantly higher than that of their paracancerous tissues (Figure 1G).

The expression of Oct4 and KPNA2 and their relationships with clinicopathological characteristics are listed in Table 1. The expression of Oct4 correlated with differentiation (*P* = 0.002) and TNM stage (*P* = 0.003), and the expression of KPNA2 correlated with histology (*P* = 0.001) and differentiation (*P* = 0.045).

### Correlation between Oct4 and KPNA2 expression in NSCLC tissues

Oct4 and KPNA2 expressions were co-localized in the same areas of the cell nuclei in some lung cancer specimens (Figure 1H–K). Of the cases studied, 25.5% showed both Oct4 and KPNA2 positive staining and 42.2% of the cases showed both Oct4 and KPNA2 negative staining. A cross analysis showed that Oct4 and KPNA2 expressions were significantly correlated (*P* = 0.000, Table 2).

**Table 2:** Oct4 and KPNA2 expression cross tabulation

**KPNA2**	**Oct4**		**Total**
	**Negative(%)**	**Positive(%)**	
negative	43(93.5)	3(6.5)	46
positive	30(53.6)	26(46.4)	56
Total	73	29	102

*P* < 0.01.

### Association with survival

**Figure 2:**
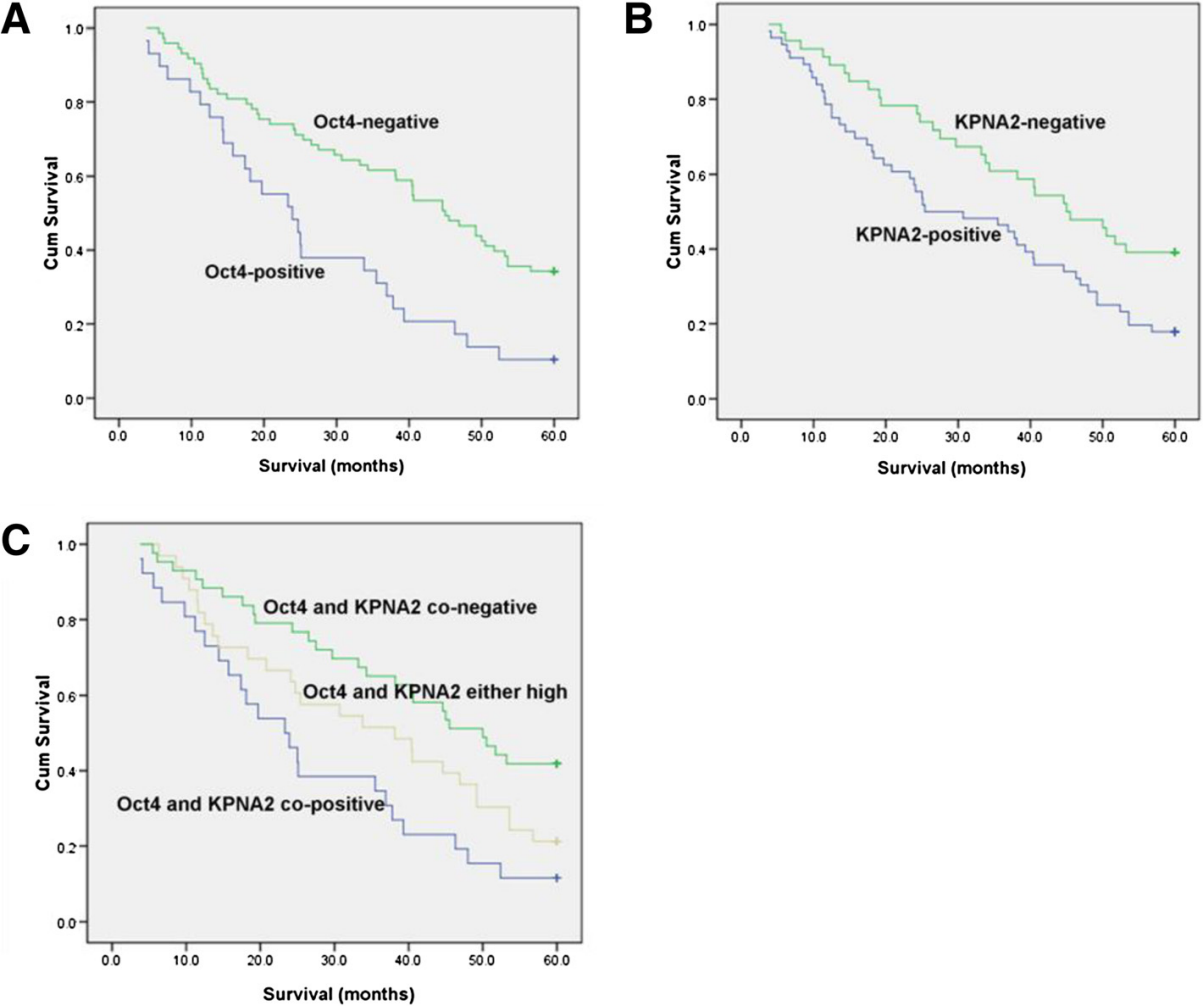
**Kaplan–Meier survival curves.** Kaplan**–**Meier curves for cumulative survival rates according to Oct4 expression status (**A**, *P* = 0.001), KPNA2 expression status (**B**, *P* = 0.013), and combined expression status of Oct4/KPNA2 (**C**, *P* = 0.003) in NSCLC. Statistical differences were calculated using log-rank comparisons.

We analyzed the relationship of Oct4 and KPNA2 expressions with the overall survival rate. A multivariate survival analysis was performed with a Cox regression model for each predictor of prognosis using the IHC results. We found that the overall survival rate was significantly lower in patients with Oct4 or KPNA2 positive expression than in patients with Oct4 or KPNA2 negative expression (*P* < 0.01, Figure 2).

### Loss of Oct4 or KPNA2 expression inhibits proliferation in tumor cells

**Figure 3:**
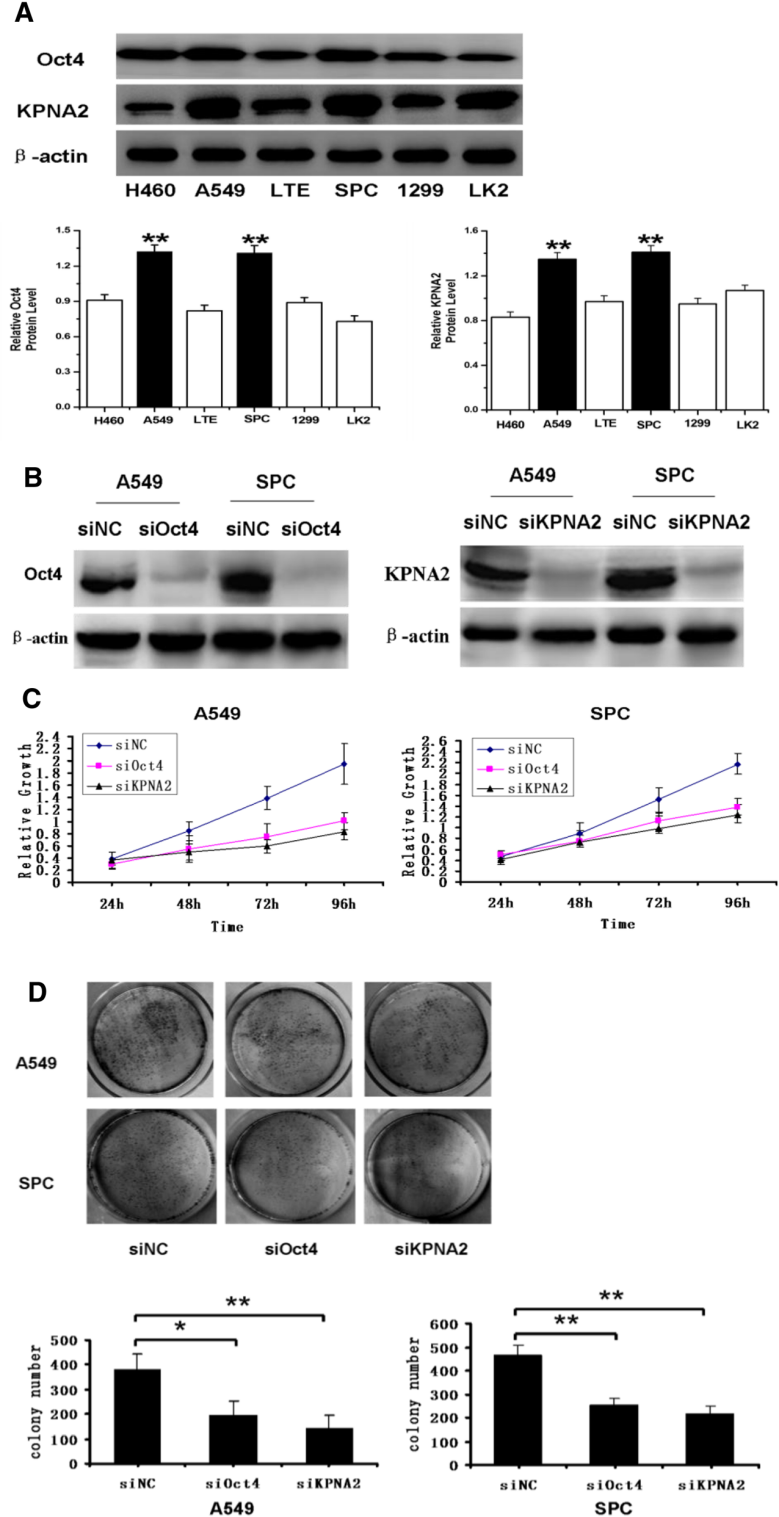
**Loss of Oct4 or KPNA2 expression inhibits proliferation in tumor cells.** Expression levels of Oct4 and KPNA2 were analyzed by western blotting in a panel of lung cancer cell lines **(A)**. Each bar represents the mean ± SD of three independent experiments. A549 and SPC cells were treated with Oct4- or KPNA2-specific siRNAs for 72 h. Western blot of Oct4 and KPNA2 depletion efficiency in A549 and SPC cell lines **(B)**. Cell growth rate was determined by the CCK-8 assay, as described in Section 2. The data are mean ± SD of three independent experiments **(C)**. (Upper panel) A549 and SPC cells transduced with siRNAs for control, Oct4, or KPNA2 were subjected to a colony formation assay. (Lower panel) Histogram quantification **(D)**. Error bars indicate ± SD.*P < 0.05, **P < 0.01.

Expression levels of Oct4 and KPNA2 were analyzed by western blotting in a panel of lung cancer cell lines (Figure 3A). The levels of Oct4 and KPNA2 were both higher in A549 and SPC cells than in the other cell lines. To explore whether Oct4 and KPNA2 play a role in cancer cell growth, we employed an RNA interference approach to knockdown Oct4 and KPNA2 expression in both A549 and SPC cell lines. The Oct4 and KPNA2 expression levels were unchanged upon transient transfection with the negative control siRNA, whereas Oct4- or KPNA2-specific siRNA significantly suppressed protein expression levels in the A549 and SPC cell lines (Figure 3B). Upon Oct4 or KPNA2 siRNA transfection, A549 cells displayed reduced growth rates compared with the negative control (Figure 3C). Similar to A549 cells, knockdown of Oct4 or KPNA2 expression also reduced the growth rate of SPC cells (Figure 3C). We used colony formation assays as an independent method to validate the antiproliferative effects of Oct4 or KPNA2 inhibition in lung cancer cells. Oct4- or KPNA2-targeting siRNAs led to a clear reduction of the colony formation capacity of A549 and SPC lung cancer cell lines compared with control siRNA-treated cells (Figure 3D). These loss-of-function studies demonstrated that the Oct4 and KPNA2 siRNAs could inhibit tumor cell proliferation compared with the control.

### Oct4 interacts with KPNA2 *in vitro*

**Figure 4:**
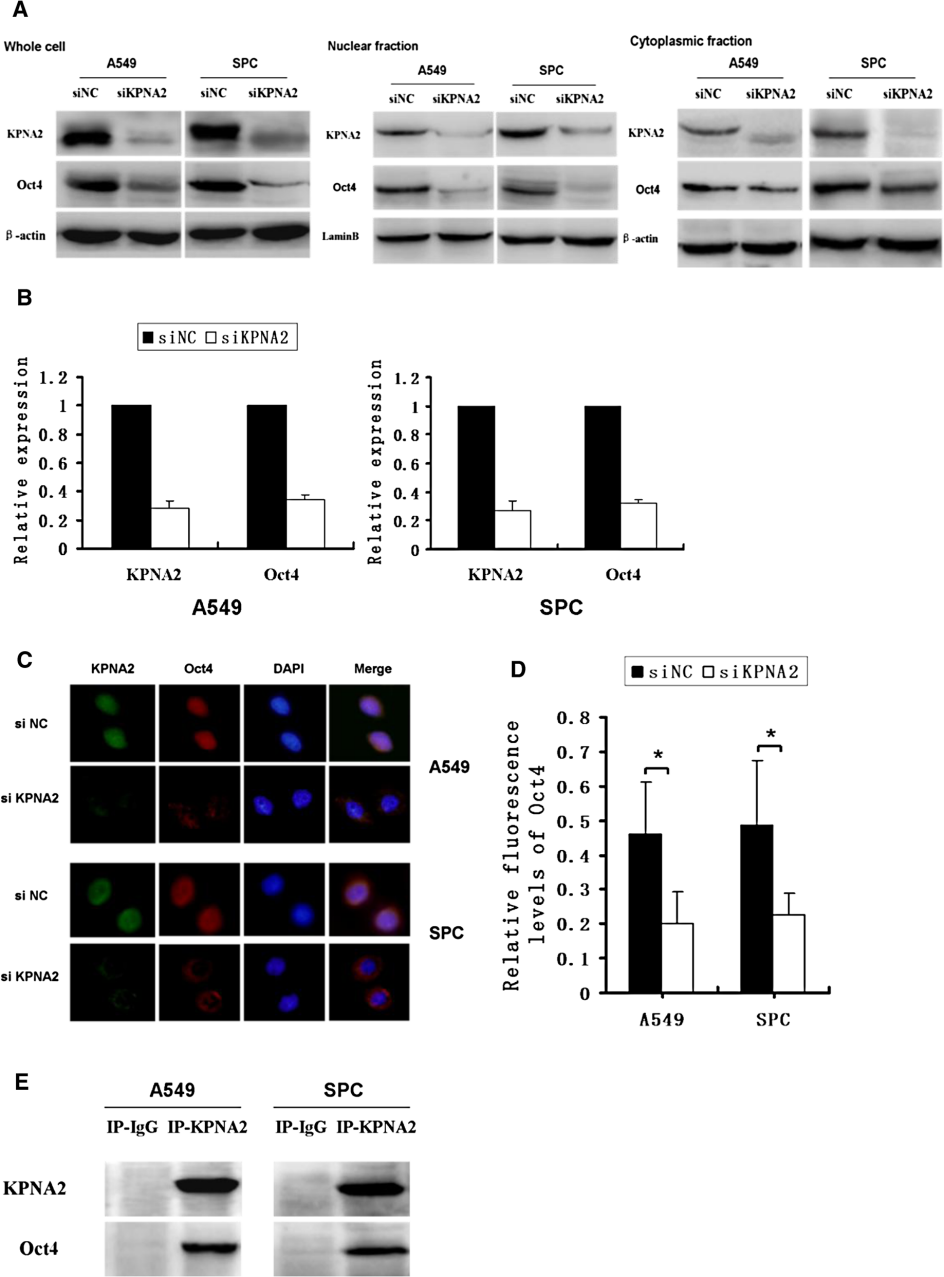
**Oct4 interacts with KPNA2 in vitro.** A549 and SPC cells were treated with KPNA2-specific siRNA for 72 h. The nucleus and cytoplasm protein expression levels of Oct4 and KPNA2 were analyzed by western blotting **(A)**. Expression of β-actin and lamin-B served as loading controls for the cytosolic and the nuclear fractions, respectively. Real-time PCR analyses of KPNA2 and Oct4 mRNA expression in cancer cells **(B)**. Immunofluorescence staining showing the localization of Oct4 and KPNA2 in A549 and SPC cancer cell lines **(C)**. Relative fluorescence levels of Oct4 were measured in cells after KPNA2 siRNA treatment. Relative fluorescence ratios of Oct4:DAPI are indicated as the mean ± SD for four fields for siNC and siKPNA2 **(D)**. *P < 0.05. Co-immunoprecipitation of KPNA2 and Oct4 in A549 and SPC cells **(E)**: whole cell lysates were immunoprecipitated with KPNA2 antibody and analyzed by western blotting with the anti-Oct4 antibody.

To explore whether KPNA2 contributes to Oct4 nuclear translocation in lung cancer, we extracted nuclear and cytoplasmic proteins, respectively, after 72 h of KPNA2 siRNA treatment with Thermo Scientific NE-PER Nuclear and Cytoplasmic Extraction Reagents. Western blotting analysis revealed that knockdown of KPNA2 decreased the nucleoprotein levels of Oct4 (Figure 4A). Furthermore, both KPNA2 and Oct4 mRNA expression levels were reduced after 72 h of KPNA2 siRNA treatment, as observed by real-time PCR (Figure 4B). Double immunofluorescence analysis was performed, and co-localization of Oct4 and KPNA2 was observed in A549 and SPC cell lines. The proportion of cells with significant nuclear Oct4 signals was reduced significantly in the KPNA2 knockdown cell group, compared with siNC cells. Cells with significant cytoplasmic Oct4 signals were observed in the KPNA2 knockdown group, but not the control group (Figure 4C-D). Co-immunoprecipitation revealed that KPNA2 interacts with Oct4 in lung cancer cell lines (Figure 4E).

## Discussion

In this study, we investigated the expression pattern and biological role of the transcription factor Oct4 and transport factor KPNA2 in 102 human NSCLC samples. Our results demonstrated that Oct4 and KPNA2 protein expression in lung cancer tissues is higher compared with corresponding normal lung tissue. We found a significant correlation between Oct4 upregulation and both differentiation and TNM stage. The expression of KPNA2 correlates with histology and differentiation. Although Oct4 and KPNA2 were expressed independently in some cases, they were more often co-localized to the same area. We also found that Oct4 and KPNA2 expressions were significantly correlated in human NSCLC tissues. This suggested that Oct4 and KPNA2 have an interaction in NSCLC. In addition, we demonstrated that Oct4 and KPNA2 upregulation correlated with a lower survival rate in NSCLC patients. This indicates that Oct4 and KPNA2 could represent novel biomarkers for distinguishing cancer from noncancerous lesions. At the same time, we found that Oct4 and KPNA2 expressions were co-positively correlated with shorter survival of NSCLC patients, compared with Oct4 and KPNA2 co-negative expression or Oct4 and KPNA2 single positive expression. Thus, combined analysis of Oct4 and KPNA2 expression in NSCLS was more informative than the analysis of either gene singly.

To validate the potential role of Oct4 and KPNA2 in lung cancer development, we employed siRNAs to knockdown Oct4 and KPNA2 expression in A549 and SPC cell lines. We found an impaired proliferation capacity and colony formation ability in both A549 and SPC cells after Oct4 or KPNA2 knockdown. These results suggest that Oct4 and KPNA2 expression were significantly correlated in lung cancer cells. High expression of these two proteins could promote the proliferation of lung cancer cells. Co-immunoprecipitation revealed that KPNA2 interacts with Oct4 in lung cancer cell lines. Moreover, knockdown of KPNA2 expression decreased the mRNA and nucleoprotein levels of Oct4. Double immunofluorescence analysis revealed that nuclear Oct4 signals were significantly reduced in the KPNA2 knockdown group.

The transcription factor Oct4 plays significant roles in maintaining pluripotency and self-renewal of embryonic stem cells and adult stem/progenitor cells anchored in a number of tissues [18,19]. Oct4 alone can induce pluripotency in mouse adult neural stem cells [20]. Furthermore, Oct4 is an important determinant for the malignant potential of tumor cells and is accordingly not expressed in healthy and differentiated tissue [21]. The Oct4 transcript can be detected in human embryonic carcinomas, testicular germ cell tumors, and seminomas [22-24]. Knocking down Oct4 in tumor-initiating cells would lead to the loss of the self-renewal and proliferating capacities and result in CSC-like apoptosis of cancer cells [25]. Our results contribute to the growing body of evidence that implicates Oct4 as a multifunctional factor involved in stem cell self-renewal and differentiation, as well as tumorigenesis and tumor progression. Recently, Oct4A was observed to contain a conserved nuclear localization signal (NLS), RKRKR, in its POU DNA binding domain [26]. It is recognized by transport receptors that carry it from the cytoplasm to the nucleus. Tumorigenesis and tumor progression are associated with dysfunction of the nuclear transport machinery.

Karyopherin-α acts as a classical NLS receptor and mammalian cells have multiple Karyopherin-α genes that are classified into three subtypes that are differentially expressed in different tissues [27-29]. A previous study indicated that KPNA2 is mainly expressed in undifferentiated ES cells [15]. Overexpression of KPNA2 caused persistent expression of pluripotency marker genes, such as Oct4, in the absence of maintenance signals, and suppressed neural differentiation [15]. Upregulation of KPNA2 expression has been implicated in several human cancers, including prostate cancer, esophageal squamous cell carcinoma, lung cancer, breast cancer, and infiltrative astrocytomas [17,30-34]. In breast tumor MCF7 cells, Oct4 expression was further increased by KPNA2 overexpression [22]. In this study, we demonstrated that knocking down KPNA2 expression decreased the mRNA and nucleoprotein levels of Oct4. This indicated that KPNA2-associated Oct4 downstream signaling may contribute to the malignant phenotype of human lung cancer cells. Oct4 transcription could be influenced by gradually varying KPNA2 expression levels. KPNA2 silencing could therefore decrease the nuclear translocation of Oct4 and suppress the proliferative capacity of lung cancer cells. Thus, Oct4 may be one of the target proteins of nuclear transport receptor KPNA2 in lung cancer cells. However, reciprocal effects between KPNA2 expression and Oct4 should be addressed in future studies.

## Conclusions

Our study demonstrated that Oct4 and KPNA2 are overexpressed in NSCLC and that this overexpression correlates with lung cancer progression. Oct4 and KPNA2 expression levels are significantly correlated. Both Oct4 and KPNA2 promote lung cancer proliferation. Oct4 nuclear localization may be mediated by its interaction with KPNA2 in lung cancer cells. KPNA2 might be a useful therapeutic target of NSCLC.

## Competing interests

The authors declare that they have no competing interests.

## Authors’ contributions

XLL and XSJ designed the research and wrote the paper. MMS, XL, and HFL collected the cases. XLL, LLJ, MMS, and XL performed the research. XLL, HFL, and ZHL analyzed the data. ZHL and EHW edited the paper. All authors read and approved the final manuscript.
